# New Appliances and Things Medical

**Published:** 1901-12-07

**Authors:** 


### NEW APPLIANCES AND THINGS MEDICAL.

**[We shall be glad to receive, at our Office, 28 & 29 Southampton Street, Strand, London, W.C., from the manufacturers, specimens of all new preparations and appliances which may be brought out from time to time.]**

### QUAKER OATS.

(Quaker Oats Company, 6 and 8 Eastcheap, London E.C.)

This special preparation of the oaten grain has from a diatetic and practical standpoint so many advantages over the old familiar oatmeal that it is scarcely necessary to mention here the particular points on which these advantages depend. However, we may call to mind the fact that ordinary ground oats, in the process of conversion into porridge, are by no means reduced to that mucilaginous condition which is the normal sequence of the rupture of the cellulose membrane, and which is practically essential for the proper digestion and assimilation of the nutritive constituents of the grain. In the case of Quaker oats the process of manufacture and the prolonged exposure to heat which that operation entails, ensures the almost complete liberation of the starch granules and the disintegration of the cellulose membrane. The oats are, therefore, presented in a form which a very little subsequent boiling reduces to a condition well adapted to our human powers of digestion. The elimination of the irritative properties of ordinary oatmeal porridge from so agreeable a breakfast dish as Quaker oats is of course of the supremest importance from a dietetic point of view. We have specially insisted on this advantage to the exclusion of the many more obvious superiorities possessed by Quaker oats when compared to the ordinary form of oatmeal, because while the latter are generally known, the former has not received the notice it deserves.

### TOILET AND OTHER SOAPS.

(Edward Cook and Co., Limited, Bow, London, E.)

The majority of Cook's soaps are too well known to require special mention. We have repeatedly examined them, and supplied our readers with the results of our investigations; in each case we have been thoroughly satisfied with the standard of purity, and the artistic merits of the manufacture. We have, too, carefully inspected the works on the occasion of a surprise visit to the Bow manufactory, and were struck with the obvious care taken in all matters of detail to ensure the incorporation in the soaps of fats, alkalies, and perfumes of only the highest quality, and in the proportions and combinations which are most approved by dermatological authorities, and best adapted for the removal of dirt or extraneous matter from the skin. Among the newer soaps we would especially mention that known as Frontier soap; it is triple-scented (brown Windsor, rose, and lavender), and particularly prepared with a view to use in the case of sensitive and delicate skins; from practical experience we can recommend this soap. During the recent fogs and cold weather, and in spite of the frequent washings which these climatic conditions entailed, the integuments of our hands remained a striking memorial to the emollient and conservative properties of this recent addition to Messrs. Cook's list of soaps. Cook's Cambath soap is also a comparatively new production. It is a camphorated, pure, skin soap for use in the bath; it has stimulating and invigorating qualities. We recommend it to the notice of householders.

### A NEW VACCINATION LYMPH.

(E. Merck, 16 Jewry Street, London, E.C.)

Messrs. Merck, of Darmstadt, have so great a reputation for care and thoroughness in all branches of chemical manufacture which are concerned in the production of medicinal and therapeutic preparations, that most of us who use and appreciate their specialities will welcome the entrance of their glycerinated calf lymph into competition with that which is produced in our own country. We understand that Merck's lymph is prepared under strict aseptic precautions, according to the method used in the Government laboratories, and it is submitted to the three following tests before it is put upon the market:—The calf is slaughtered after removal of the lymph, and carefully examined as to the state of its internal organ; if not healthy, the lymph is rejected. Secondly, the lymph is examined bacteriologically for the presence of extraneous and pathogenic bacteria. Thirdly, the lymph is tested for efficiency by test examination. We have put the lymph to practical test, and find it entirely satisfactory and energetic in action. Each tube is most carefully packed, and protected from the influence of light.

